# Licoricesaponin G2 ameliorates bleomycin-induced pulmonary fibrosis via targeting TNF-α signaling pathway and inhibiting the epithelial-mesenchymal transition

**DOI:** 10.3389/fphar.2024.1437231

**Published:** 2024-09-05

**Authors:** Jing Ma, Lu Ding, Xiaoyu Zang, Ruonan Wei, Yingying Yang, Wei Zhang, Hang Su, Xueyan Li, Min Li, Jun Sun, Zepeng Zhang, Zeyu Wang, Daqing Zhao, Xiangyan Li, Linhua Zhao, Xiaolin Tong

**Affiliations:** ^1^ College of Traditional Chinese Medicine, Changchun University of Chinese Medicine, Changchun, China; ^2^ Key Laboratory of Active Substances and Biological Mechanisms of Ginseng Efficacy, Jilin Provincial Key Laboratory of Bio-Macromolecules of Chinese Medicine, Ministry of Education, Northeast Asia Research Institute of Traditional Chinese Medicine, Changchun University of Chinese Medicine, Changchun, China; ^3^ Research Center of Traditional Chinese Medicine, College of Traditional Chinese Medicine, Changchun University of Chinese Medicine, Changchun, China; ^4^ Shiyan Hospital of Traditional Chinese Medicine, Shiyan, China; ^5^ China-Japan Friendship Hospital, National Center for Integrated Traditional Chinese and Western Medicine, Beijing, China; ^6^ School of Basic Medicine, Gansu University of Traditional Chinese Medicine, Lanzhou, China; ^7^ College of Integrated Traditional Chinese and Western Medicine, Changchun University of Chinese Medicine, Changchun, China; ^8^ Institute of Metabolic Diseases, Guang’ Anmen Hospital, China Academy of Chinese Medical Sciences, Beijing, China

**Keywords:** licoricesaponin G2, pulmonary fibrosis, epithelial-mesenchymal transition, TNF-α signaling pathway, network analysis

## Abstract

**Background:**

Pulmonary fibrosis (PF) emerges as a significant pulmonary sequelae in the convalescent phase of coronavirus disease 2019 (COVID-19), with current strategies neither specifically preventive nor therapeutic. Licoricesaponin G2 (LG2) displays a spectrum of natural activities, including antibacterial, anti-inflammatory, and antioxidant properties, and has been effectively used in treating various respiratory conditions. However, the potential protective effects of LG2 against PF remain underexplored.

**Methods:**

Network analysis and molecular docking were conducted in combination to identify the core targets and pathways through which LG2 acts against PF. In the model of bleomycin (BLM)-induced C57 mice and transforming growth factor-β1 (TGF-β1)-induced A549 and MRC5 cells, techniques such as western blot (WB), quantitative Real-Time PCR (qPCR), Immunohistochemistry (IHC), Immunofluorescence (IF), and Transwell migration assays were utilized to analyze the expression of Epithelial-mesenchymal transition (EMT) and inflammation proteins. Based on the analysis above, we identified targets and potential mechanisms underlying LG2’s effects against PF.

**Results:**

Network analysis has suggested that the mechanism by which LG2 combats PF may involve the TNF-α pathway. Molecular docking studies have demonstrated a high binding affinity of LG2 to TNF-α and MMP9. Observations from the study indicated that LG2 may mitigate PF by modulating EMT and extracellular matrix (ECM) remodeling. It is proposed that the therapeutic effect is likely arises from the inhibition of inflammatory expression through regulation of the TNF-α pathway.

**Conclusion:**

LG2 mitigates PF by suppressing TNF-α signaling pathway activation, modulating EMT, and remodeling the ECM. These results provide compelling evidence supporting the use of LG2 as a potential natural therapeutic agent for PF in clinical trials.

## Introduction

PF represents a severe form of interstitial lung disease that progressively impairs lung function, as evidenced by increasing morbidity and mortality rates with age (Raghu et al., 2015; Wynn, 2011). Characterized by abnormal proliferation and damage of alveolar epithelial cells, excessive deposition of ECM, and the activation and proliferation of fibroblasts (Rockey et al., 2015; Sgalla et al., 2018), this chronic condition typically follows an irreversible pathological course (Richeldi et al., 2017). Despite ongoing research, the precise pathogenesis of PF remains unclear, and it is associated with a low average survival rate of only 2.8 years post-diagnosis. The disease’s lethality has led to its description as a “tumor-like disease.” Currently, pirfenidone and nintedanib are the only two drugs clinically proven to possess anti-PF properties, shown to enhance respiratory function in patients with PF (Flaherty et al., 2019; King et al., 2014). However, these treatments only slow the disease progression and cannot stop it completely. Common side effects include gastrointestinal, kidney, and liver discomfort (Maher et al., 2020). Additionally, the high costs and limited efficacy of these treatments, combined with significant adverse effects, highlight the pressing need for more effective, tolerable, and affordable therapeutic options for PF. In recent years, traditional Chinese medicine (TCM) have demonstrated protective effects against lung injury. For instance, an observational study revealed that the recurrence rate of positive RT-PCR test results was significantly lower with comprehensive TCM interventions, at 2.8%, compared to 15.8% without such interventions. These findings suggest that comprehensive TCM intervention is an independent factor influencing nucleic acid recurrence (He et al., 2020). Isoliquiritin apioside, a flavonoid from Glycyrrhiza uralensis Fisch (Gancao), exhibited antioxidant and anti-inflammatory properties that alleviated acute lung injury by inhibiting HIF-1α-induced ferroptosis (Zhongyin et al., 2022). Furthermore, total flavonoids from Astragalus membranaceus (Fisch.) Bunge (Huangqi) have shown efficacy in reducing BLM-induced PF in mice by modulating inflammatory cytokine expression and M2 macrophage polarization (Yang et al., 2023).

Epithelial-mesenchymal transition (EMT), a dynamic and reversible process, is essential in embryonic development, wound healing, and fibrosis. Upon tissue injury or pathological stimuli, epithelial cells may undergo EMT, thereby losing epithelial characteristics and acquiring increased mobility, invasiveness, and expression of myofibroblast markers such as N-cadherin, α-SMA, and Collagen I (Peng et al., 2020). This transformation also involves a reduction in epithelial markers like E-cadherin. Ideally, a dynamic equilibrium between extracellular matrix (ECM) synthesis and degradation preserves lung tissue homeostasis, but excessive ECM accumulation can lead to scarring and lung tissue destruction. Consequently, modulating EMT and ECM deposition emerges as a potential therapeutic approach to prevent pulmonary fibrosis (PF). Thus, investigating the regulatory mechanisms of EMT and ECM in PF to identify effective natural active products is of paramount importance.

Glycyrrhiza uralensis Fisch [commonly known as Gancao, abbreviated as GC; verified via “World Flora Online” (http://www.worldfloraonline.org/taxon/wfo-0000186028, accessed on 17 Oct 2023)] is a well-established natural medicine. Historically, GC was first documented in “Shennong’s Classic of Materia Medica.” Subsequent records emphasize its ample resources and varied uses. Notably, about one-third of over 96,000 TCM formulas incorporate GC ( Guo et al., 2014). According to TCM principles, GC primarily provides antipyretic, detoxifying, phlegm-dissolving, and antitussive benefits. Presently, GC is extensively utilized to manage various respiratory ailments, including COVID-19 (Xiong et al., 2020), acute lung injury (Zhongyin et al., 2022), PF (Ding et al., 2023; Wang et al., 2021), and asthma (He et al., 2023). Recent pharmacological studies have identified that natural bioactive metabolites in GC, such as glycyrrhetinic acid, demonstrate dose-dependent antitussive and expectorant effects. Moreover, glycyrrhetinic acid and glycyrrhizin exhibit significant antipyretic properties (Xiong et al., 2020).

**FIGURE 1:**
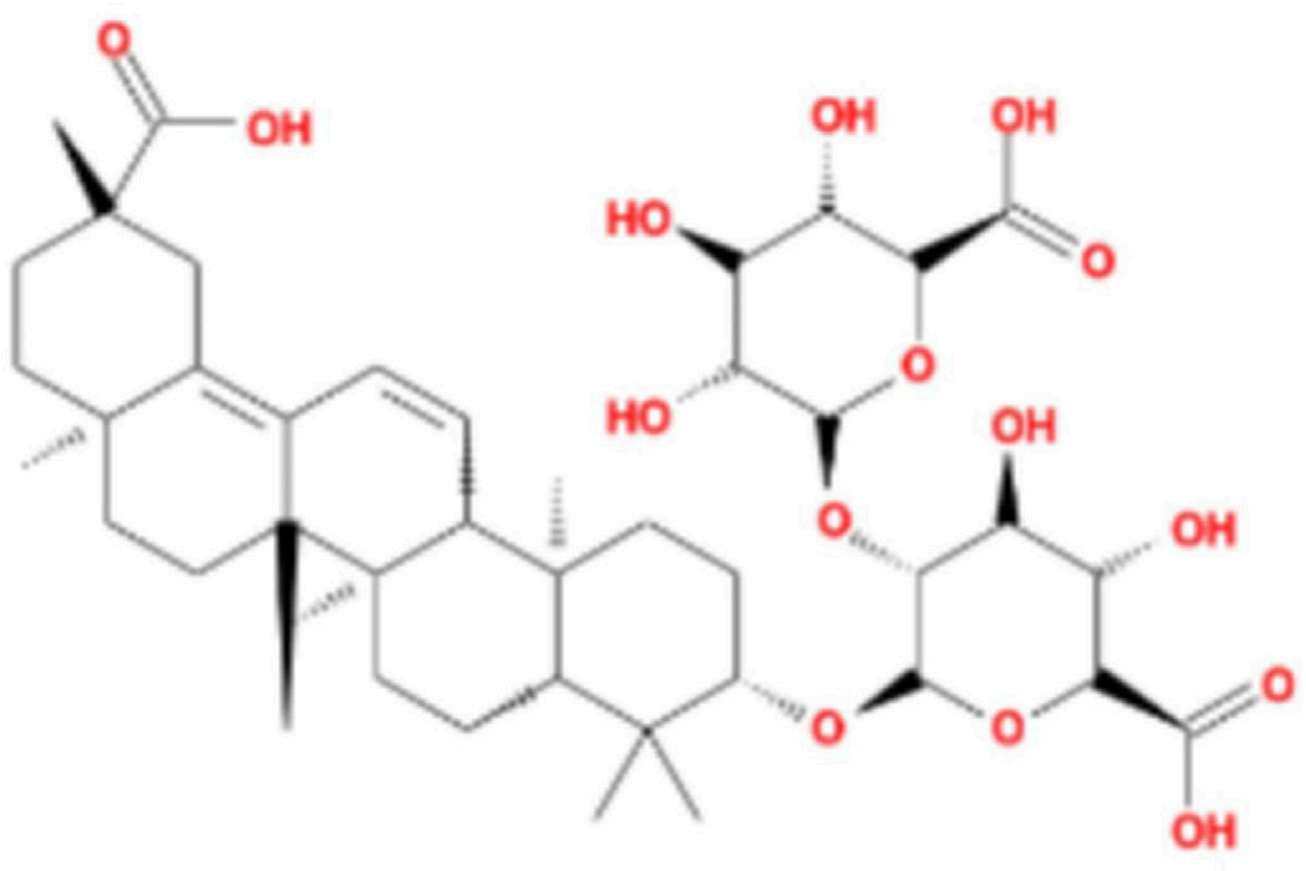
2D structure of LG2.

LG2 (Figure 1), a pentacyclic triterpenoid sourced from the rhizome of Glycyrrhiza uralensis Fisch, was selected based on our team’s previous research on the anti- PF effects of Qimai Feiluoping Decoction. UHPLC/IM-QTOF-MS identified ten bioactive metabolites, seven of which are derivatives from GC, such as liquiritin, isoliquiritigenin, neoisoliquiritin, licoricesaponin A3, licoricesaponin G2, and licoricesaponin K2. Molecular docking studies revealed the binding energies of these metabolites with TGF-β1 and TGF-βR1. Remarkably, LG2 showed the highest binding energies, with values of −8.2 kcal/mol for TGF-β1 and −7.8 kcal/mol for TGF-βR1 (Yang et al., 2021). These findings designate LG2 as a promising candidate in natural phytomedicine for combating PF, though further research is necessary to pinpoint the specific metabolites contributing to its anti-PF properties. To address the existing gaps in this field and enhance the novelty and innovation of our research focus, LG2 has been selected for detailed analysis in this study.

**FIGURE 2:**
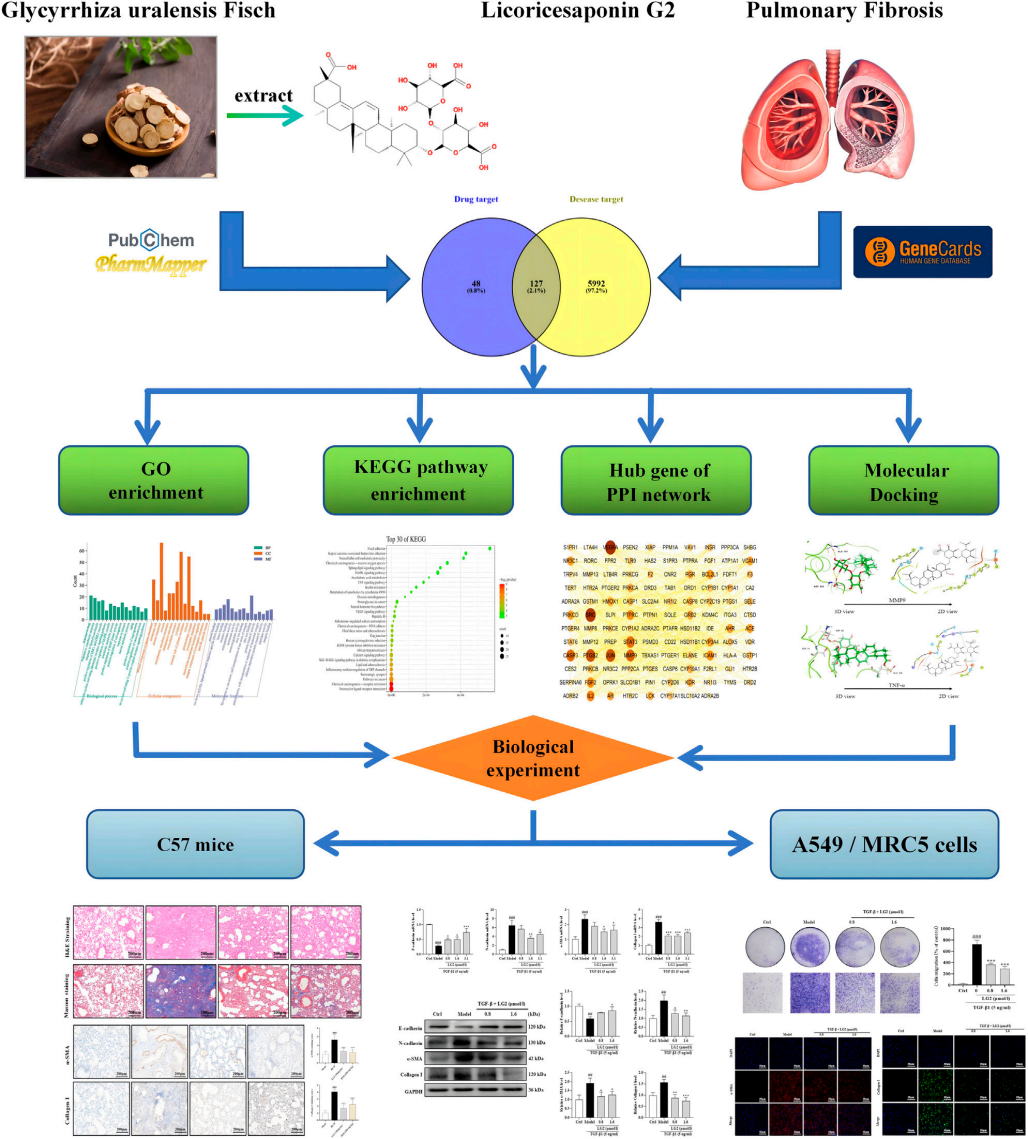
The flow chart of this research.

Network analysis integrates biological networks with drug action networks to analyze the interrelationships among components and diseases. This novel approach enlightens potential pathways through which TCMs may exert their effects, thereby enhancing our understanding of their mechanisms in disease treatment. Molecular docking, conversely, simulates geometric structures and molecular interactions to inform drug design. Recently, the integration of these two methods has emerged as a significant trend in drug research and discovery. Compared to standalone biological experiments, the efficacy of TCM, evaluated through network analysis coupled with experimental validation, can be understood more comprehensively and objectively. In this study, we employed network analysis and molecular docking to delineate the core “disease-targets-pathways” through which LG2 exerts its anti-PF effects. Furthermore, to explore the efficacy and potential mechanisms of the natural product LG2 in depth, experimental verification was conducted using BLM-induced C57 mice and TGF-β1-induced A549, MRC5 cells *in vitro* and *in vivo*. The research flowchart is depicted in Figure 2.

## Materials and methods

### Reagents and antibodies

Licoricesaponin G2 (CAS: 118441-84-2, purity >98%) was sourced from Sichuan Weikeqi Biotechnology Co., Ltd., Sichuan, China. Bleomycin (BLM, HY17565), pirfenidone (HYB0673), TGF-β1 (HY-151427) and Etanercept (HY-108847) were acquired from MedChemExpress, Princeton, United States. RPMI 1640 medium and fetal bovine serum were procured from Gibco, United States, and Clark Bioscience, Claymont, United States, respectively. Minimum Essential Medium was obtained from Procell, Wuhan, China. Paraformaldehyde (4%) and penicillin-streptomycin were supplied by Biosharp, Hefei, China. MTT and Triton X-100 were purchased from Solibol, Beijing, China, while DAPI was sourced from Solibo, Beijing, China. The Magnetic Cell Total RNA Kit and iScript cDNA synthesis kit were procured from Tiangen Biochemical Technology Co., Ltd., Beijing, China. RIPA buffer was obtained from Beyotime Biotechnology, Shanghai, China. PVDF membranes were sourced from Roche, Basel, Switzerland. The Transwell apparatus (8-μm pore size) was purchased from Corning Costar, Cambridge, United States. Antibodies against E-cadherin (ab40772), N-cadherin (ab76011), and GAPDH (ab8245) were acquired from Abcam, Cambridge, United Kingdom. Antibodies against α-SMA (#AF1032) were sourced from Affinity Biosciences, Jiangsu, China. Antibodies against Collagen I (14695-1-AP), TNF-α (CL488-60291), MMP3 (66338-1-Ig), MMP9 (10375-2-AP), β-actin (66009-1-Ig-AP), and goat anti-rabbit/mouse antibodies (SA00001-1, SA00001-2) were obtained from Proteintech, Wuhan, China. p-IKK alpha/beta (BZ16321), NFκB (AP0076), and p-NFκB (BS66162) were purchased from Bioworld, Nanjing, China.

### Screening active ingredient targets of LG2

Potential targets for LG2 were identified using several databases, including Drugbank, Swisstarget, Prediction, ZINC, and PharmMapper. After consolidating the data from these sources, duplicate values were eliminated to derive the unique potential targets of LG2.

### Collection of target genes related to PF

Using “Pulmonary Fibrosis” as the search keyword, target genes associated with PF were extracted from three reputable databases: GeneCards (https://www.genecards.org/), Online Mendelian Inheritance in Man (OMIM) (https://omim.org/), and PharmGKB (https://www.pharmgkb.org/). These databases are widely recognized as reliable sources for obtaining disease-related targets.

### Acquisition of genes in the intersection between PF and LG2

A Venn diagram was utilized to illustrate the overlapping regions among sets of elements. Initially, the principal target genes associated with both PF and LG2 were input into Venny 2.1 (http://bioinfogp.cnb.csic.es/tools/venny/). Subsequently, the intersecting targets of LG2 and PF were identified using the same tool. This analysis ultimately identified the potential target genes of LG2 relevant to the treatment of PF.

### Construction of protein-protein interaction network

A protein-protein interaction (PPI) network was established using the STRING database (https://string-db.org/) (Szklarczyk et al., 2019). Initially, the overlapping targets of LG2 and PF were inputted into the STRING database to create a preliminary PPI network diagram. Subsequently, the species was set to “*Homo sapiens*,” and isolated proteins were excluded. An enhanced PPI network diagram was then developed using Cytoscape software (version 3.7.2). Within the Cytoscape software, the “Network Analyzer” feature was utilized to conduct an analysis. Key metrics, such as betweenness centrality (BC), closeness centrality (CC), and degree centrality (DC), were selected as screening indices. These degrees were sorted in descending order. Following a comprehensive review of the literature, pivotal targets were identified.

### GO analysis and KEGG pathway enrichment

To explore the signaling pathways and potential biological processes involved in LG2 treatment of PF, we entered the intersecting targets of LG2 and PF into the DAVID database (https://david.ncifcrf.gov/) (Dennis et al., 2003). Within the “Functional Annotation” section, we selected CC, molecular functions (MF), and biological processes (BP) under Gene Ontology (GO), along with the Kyoto Encyclopedia of Genes and Genomes (KEGG) pathways. The results were downloaded and saved. Then, these results were filtered using Excel, retaining only those with *p*-values <0.05 and counts ≥5. Using the bioinformatics platform, the top 30 KEGG pathway enrichment results were presented in a bubble diagram and the top 15 GO enrichment results in a bar chart, based on gene number and *p*-value. Following a comprehensive literature review, a specific pathway was selected for further investigation.

### Molecular docking of active ingredients with key targets

The Protein Data Bank (PDB) (Protein Data Bank, 2019) is the premier database for the three-dimensional structural data of proteins, nucleic acids, and other biological macromolecules. These structures are primarily determined through experimental techniques such as X-ray crystallography, nuclear magnetic resonance (NMR), and electron microscopy. In our study, we selected two targets as receptors and LG2 for molecular docking validation. Initially, the crystal structure of the target protein was retrieved from the PDB database. Any bound ligand structures were removed, and the ligand binding site or the active site, as identified in the literature, was designated as the binding pocket for the small molecule ligand. Subsequently, after preprocessing the target protein using the PrepWiz module of Schrodinger software, a grid file was generated. Concurrently, details such as the Mol ID number, CAS number, names in both Chinese and English, and the chemical 2D structure of LG2 were obtained from the Traditional Chinese Medicine Systems Pharmacology (TCMSP) database and saved in the mol.2 format. LG2 was processed using the LigPrep module of Schrodinger software. For the final step, the prepared ligand-receptor file underwent molecular docking using the Standard Precision method in Schrodinger’s Glide module. The ionization state was assigned using Epik28 under pH conditions of 7.0 ± 2.0, followed by docking calculations. A default binding score of less than −5.0 kcal/mol indicates favorable binding activity between the ligand and receptor, while a score below −7.0 kcal/mol suggests particularly strong binding affinity (Lipinski et al., 2001).

### Animal experiment design

The experimental protocol involving animals was approved by the Experimental Animal Ethics Committee of Changchun University of Chinese Medicine (Approval No. 2023608). We acquired 8-week-old male C57/BL6 mice from Beijing Vital River Laboratory Animal Technology Co., Ltd., Beijing, China. These mice were maintained under optimal conditions in our animal experiment center at 20°C ± 2°C with 50%–60% humidity. Currently, transtracheal administration is recognized as the more mature method. High-quality research has established typical dosages for BLM-induced PF in mice are 3, 5, and 10 mg/kg (Lee et al., 2020). Based on this, our team explored the pre-experimental conditions using different dosages. We found that although 5 mg/kg and 10 mg/kg could induce PF as well, the modeling mice were in poorer health and higher mortality rates. Therefore, we ultimately chosed 3 mg/kg (Ding et al., 2023) as the optimal dosage to reliably model the onset of PF (the success rate of animal modeling is over 93%). Pirfenidone was selected as the positive control. The mice were randomly assigned to four groups: sham operation, bleomycin (BLM), LG2 (oral gavage: 50 mg/kg), and pirfenidone (PFD, oral gavage: 200 mg/kg), with each group consisting of 10 mice. All mice received an intraperitoneal injection of pentobarbital sodium (30 mg/kg) for anesthesia. The sham operation group was administered an intra-tracheal injection of 0.9% saline (NS), while the other groups received BLM (3 mg/kg). The mice underwent daily oral administration of LG2, with the sham and model groups receiving pure water for 2 weeks, followed by euthanasia and tissue collection for further experimental analysis. The layout and design of the animal experiment are depicted in Figure 5A.

### Cell culture and treatment

A549 cells (human lung carcinoma) and MRC5 cells (human fetal lung fibroblasts) were obtained from the Cell Bank of Cell Biology at the Chinese Academy (Shanghai, China) and Fu Heng Biology (Shanghai, China), respectively. A549 cells were cultured in RPMI 1640 medium, while MRC5 cells were maintained in Minimum Essential Medium. Both media were supplemented with 10% fetal bovine serum (FBS) and 1% antibiotics (penicillin and streptomycin). The cells were divided into five groups: a control group, a model group treated with TGF-β1 at 5 ng/mL, and three groups exposed to varying concentrations of TGF-β1. To induce EMT, TGF-β1 was added to the cultures (except the control group) upon reaching 60%–70% confluence, and the co-culture was continued for 48 h. All cell lines were incubated at 37°C in a 5% CO₂ atmosphere.

### Histopathology and immunohistochemistry

The lung lobes were washed with chilled saline before fixation in 4% paraformaldehyde. The tissues were then embedded in paraffin and sectioned into 3 μm slices, which were subsequently stained with H&E and Masson’s trichrome for detailed histological analysis. The stained sections were meticulously examined using a digital microscope and slide scanner (M8, PreciPoint, Thuringia, Germany). Pathological scoring was conducted based on methodologies described in prior studies (Katoh et al., 1998; Pedroza et al., 2016). Concurrently, to assess the expression levels of proteins related to EMT, ECM, and IHC staining was performed to detect biomarkers including α-SMA, Collagen I, TNF-α, and MMP9. Following dewaxing and antigen retrieval, the tissues were incubated overnight at 4°C with primary antibodies, followed by a 1-h incubation with HRP-labeled secondary antibodies. DAB solution was applied to develop brown staining, and a final counterstain with hematoxylin was performed. The M8 was used to select appropriate fields of view for quantitative analysis of the pathological findings using ImageJ software.

### Immunofluorescence

Dewaxed lung tissue sections and treated A549 cells were incubated overnight with primary antibodies, including α-SMA, Collagen I, TNF-α, and MMP9. This was followed by a 1 h incubation with corresponding secondary antibodies at room temperature. Nuclei were stained using DAPI for 15 min. Finally, relevant fields of view were photographed using a fluorescence microscope (Leica DMi8, Germany), and fluorescence intensity was quantified using ImageJ software.

### Western blot analysis

Proteins were extracted from lung tissues and A549 and MRC5 cells using pre-cooled RIPA lysis buffer. The protein concentration was determined using a BCA protein assay. The proteins of interest were then separated by SDS-PAGE and transferred onto PVDF membranes. After blocking for 1 h, the membranes were incubated overnight at 4°C with primary antibodies against E-cadherin, N-cadherin, α-SMA, Collagen I, TNF-α, p-IKK, p-NFκB, NFκB, MMP3, MMP9, β-actin, and GAPDH. Following washing with 1× TBST, the membranes were treated with secondary antibodies for 1 h at room temperature. Protein bands were visualized using a chemiluminescent imaging system (ChemiDoc XRS+, Bio-Rad), and quantification was performed using ImageJ software.

### CCK8 assay for cell viability

A549 and MRC5 cells were seeded in a 96-well plate at a density of 5,000 cells per well. These cells were treated with various concentrations of LG2, ranging from 0.2 to 50 μmol/L, for 48 h to evaluate the metabolite’s effect on cell viability. Additionally, to assess the impact of LG2 on TGF-β1-treated cells (5 ng/mL), A549 and MRC5 cells were divided into control, model, and treatment groups. After 48 h of incubation, MTT assay was conducted by adding 0.5 mg/mL MTT to each well and incubating for 4 h at 37°C in the dark. Subsequently, 150 μL of DMSO was added to dissolve the formazan crystals. Absorbance was measured at a wavelength of 490 nm using a microplate reader (American Bertten, SynergyMx).

### Quantitative real-time PCR analysis

Total RNA was extracted from A549 and MRC5 cells using a total RNA extraction kit. Subsequently, 1 μg of total RNA was reverse transcribed into cDNA utilizing an iScript cDNA synthesis kit. qPCR analysis was conducted on the Bio-Rad CFX96 system (Bio-Rad, Synergy Mx). Data normalization was performed using the 2^−ΔΔCt^ method with GAPDH as the reference gene (Yang et al., 2021). The primer sequences used are detailed in Table 1.

**TABLE 1:** Primer sequences for quantitative real-time PCR analysis.

Primer	Sequence (5′-3′)
(Human) E-cadherin-F	CGA​GAG​CTA​CAC​GTT​CAC​GG
(Human) E-cadherin-R	GGG​TGT​CGA​GGG​AAA​AAT​AGG
(Human) N-cadherin-F	TCA​GGC​GTC​TGT​AGA​GGC​TT
(Human) N-cadherin-R	ATG​CAC​ATC​CTT​CGA​TAA​GAC​TG
(Human) α-SMA-F	TAG​CAC​CCA​GCA​CCA​TGA​AG
(Human) α-SMA-R	CTG​CTG​GAA​GGT​GGA​CAG​AG
(Human) Collagen 1-F	TTC​TGC​AAC​ATG​GAG​ACT​GG
(Human) Collagen 1-R	AAT​CCA​TCG​GTC​ATG​CTC​TC
(Human) GAPDH-F	CAC​CCA​CTC​CTC​CAC​CTT​TG
(Human) GAPDH-R	CCA​CCA​CCC​TGT​TGC​TGT​AG

### Transwell migration assays

The migration of A549 cells was assessed using Transwell Boyden chambers equipped with polycarbonate filters containing 8 μm pores. Cells were seeded into the upper chamber pre-coated with Matrigel. These chambers were then placed in 24-well plates and incubated for 24 h in a serum-free medium, treated either with TGF-β1 alone or in combination with LG2. After the incubation, the medium in the upper chamber was removed. The cells were fixed with 4% paraformaldehyde for 1 h. Non-migrated cells on the filter side of the upper chamber were removed using a cotton swab. Following fixation, cells that had migrated to the bottom surface of the membrane were washed three times with PBS and stained with 5% Cresyl violet for 15 min. The migrated cells were then photographed under a microscope for analysis.

### Statistical analysis

The experimental results were expressed as the mean ± standard deviation, based on at least three independent experiments. Data analysis was performed using GraphPad Prism version 9.0. To assess statistical significance among multiple groups, one-way ANOVA followed by Tukey’s *post hoc* test was utilized. In all statistical analyses, a *p*-value of less than 0.05 was considered to indicate statistical significance.

## Results

### Screening active ingredient targets of LG2

After screening, there are 175 potential targets of LG2 were predicted by Durgbank, Swisstarget, Prediction, ZINC and PharmMapper.

### Collection of gene targets associated with PF

The keywords “Pulmonary fibrosis” were used in GeneCards, OMIM, and PharmGKB databases for PF disease targets were searched and integrated to obtain PF-related targets after removing duplicate targets. The three databases of GeneCards, OMIM, and PharmGKB were searched to obtain 6,119 PF disease targets.

### Acquisition of genes in the intersection between PF and LG2

**FIGURE 3:**
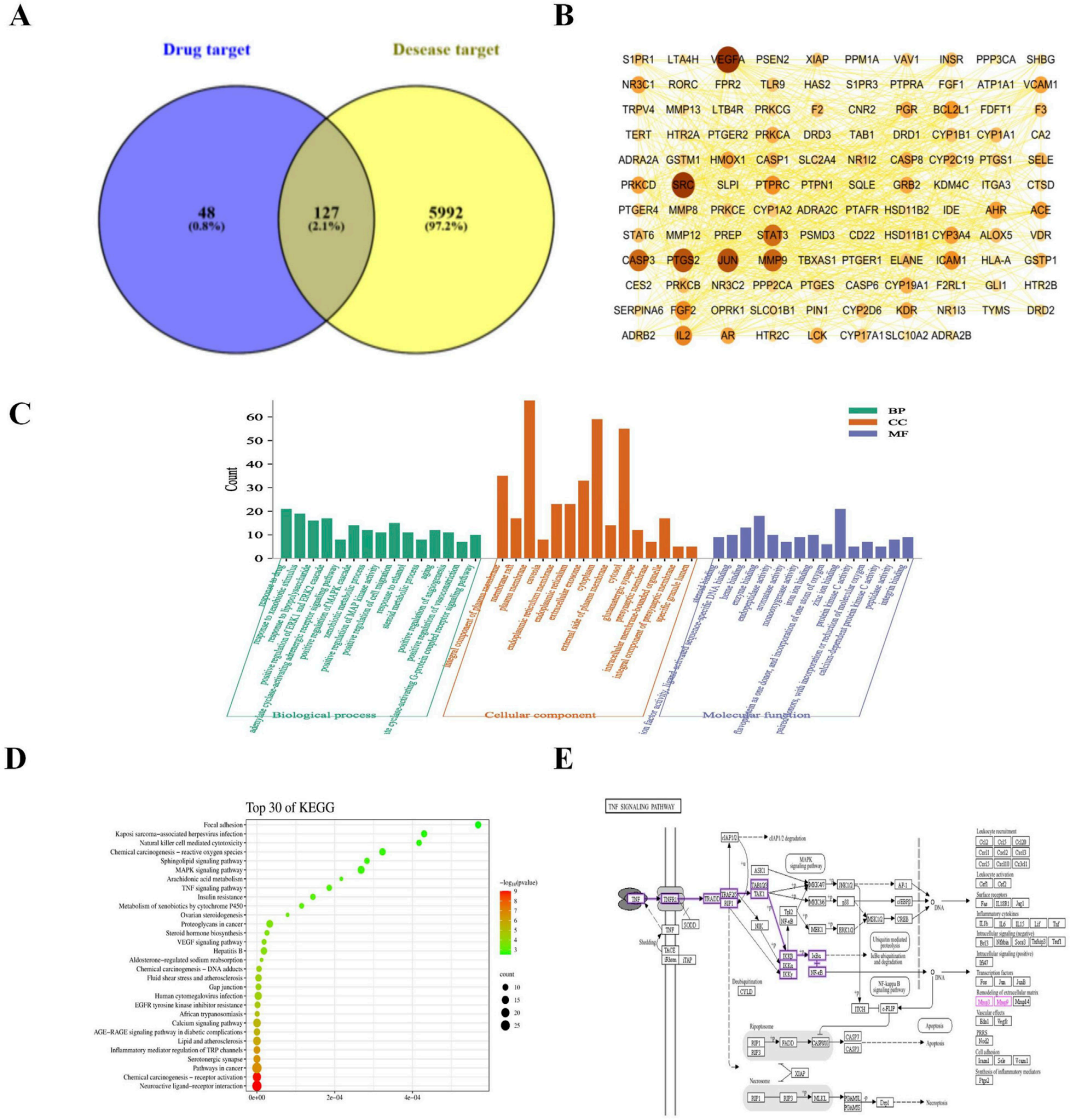
**(A)** Venn diagram depicting potential targets for PF treatment with LG2. **(B)** Advanced protein-protein interaction (PPI) network of LG2. **(C)** Analysis of gene ontology (GO) biological processes for LG2’s active ingredient. **(D)** Enrichment analysis of active ingredient-target KEGG pathways. **(E)** TNF-α signaling pathway extracted from KEGG.

Venn analysis of the 6,119 disease targets and 175 drug targets revealed 127 potential targets for LG2 in treating PF, as depicted in Figure 3A.

### Protein interactions network construction

Utilizing the STRING database’s multiple proteins tool, we uploaded the intersecting targets while specifying *H. sapiens* as the species. We set the confidence level at greater than 0.9 and excluded disconnected nodes to generate the PPI network. The data, in TSV format, was downloaded and imported into Cytoscape 3.7.2 to visualize a refined PPI network, as illustrated in Figure 3B. This network comprised 118 nodes and 797 edges. Node size and color intensity were determined based on degree values, with larger and darker nodes indicating higher degrees. Edge color intensity was set based on the relationship value score between target points, with darker edges indicating higher scores. Topological analysis was conducted using the Network Analyzer tool in Cytoscape. Median values for BC, CC, and degree centrality (DC) were calculated as 0.00326225, 0.439025935, and 10, respectively. Targets meeting the median for all three metrics were filtered and ranked by degree value in descending order. The top 10 targets, based on a composite ranking of BC, CC, and DC, were identified as VEGFA, SRC, PTGS2, JUN, MMP9, CASP3, STAT3, IL2, FGF2, and PTPRC.

### GO function and KEGG pathway enrichment analysis

The GO enrichment analysis revealed that among 421 biological process pathways, 111 (with *p* < 0.05 and count ≥5) were predominantly associated with responses such as “response to drug,” “response to xenobiotic stimulus,” “response to lipopolysaccharide,” as well as the “positive regulation of the ERK1 and ERK2 cascade” and the “positive regulation of the MAPK cascade.” In the category of cellular components, 38 out of 62 pathways (with *p* < 0.05 and count ≥5) were primarily linked to “membrane raft,” “integral component of plasma membrane,” “plasma membrane,” “caveola,” and “endoplasmic reticulum.” Regarding molecular functions, 42 out of 99 pathways (with *p* < 0.05 and count ≥5) were chiefly related to “steroid binding,” “heme binding,” and “enzyme binding.” These findings are illustrated in Figure 3C. The KEGG pathway analysis indicated that of the 106 pathways associated with PF, 80 (with *p* < 0.05 and count ≥5) primarily pertained to “Neuroactive ligand-receptor interaction,” “TNF-α signaling pathway,” “MAPK signaling pathway,” and “EGFR tyrosine kinase inhibitor resistance,” as depicted in Figure 3D. It is well-established that inflammation plays a pivotal role in the progression of PF. Exposure to harmful stimuli can trigger tissue apoptosis and necrosis through inflammatory infiltration. The aggregation of fibroblasts may lead to significant ECM deposition, increased fibrous tissue, and ultimately restricted respiratory function in lung tissues, progressing to respiratory failure. Tumor necrosis factor (TNF-α) receptors, ubiquitously expressed across cells and tissues, have broad and multifaceted roles in inflammatory responses and can either induce or exacerbate tissue fibrosis. Furthermore, TNF-α can diminish the activity of matrix metalloproteinases (MMPs), undermining ECM decomposition by MMPs and exacerbating ECM accumulation. Through network analysis, the TNF-α signaling pathway was identified as shown in Figure 3E. This aligns with the inflammatory perspective, prompting an investigation into whether LG2 could mitigate the onset of EMT/fibroblast myofibroblast transition (FMT), enhance ECM degradation, reduce ECM deposition, and ultimately alleviate PF by suppressing the expression of the TNF-α pathway.

### Molecular docking validation

**FIGURE 4:**
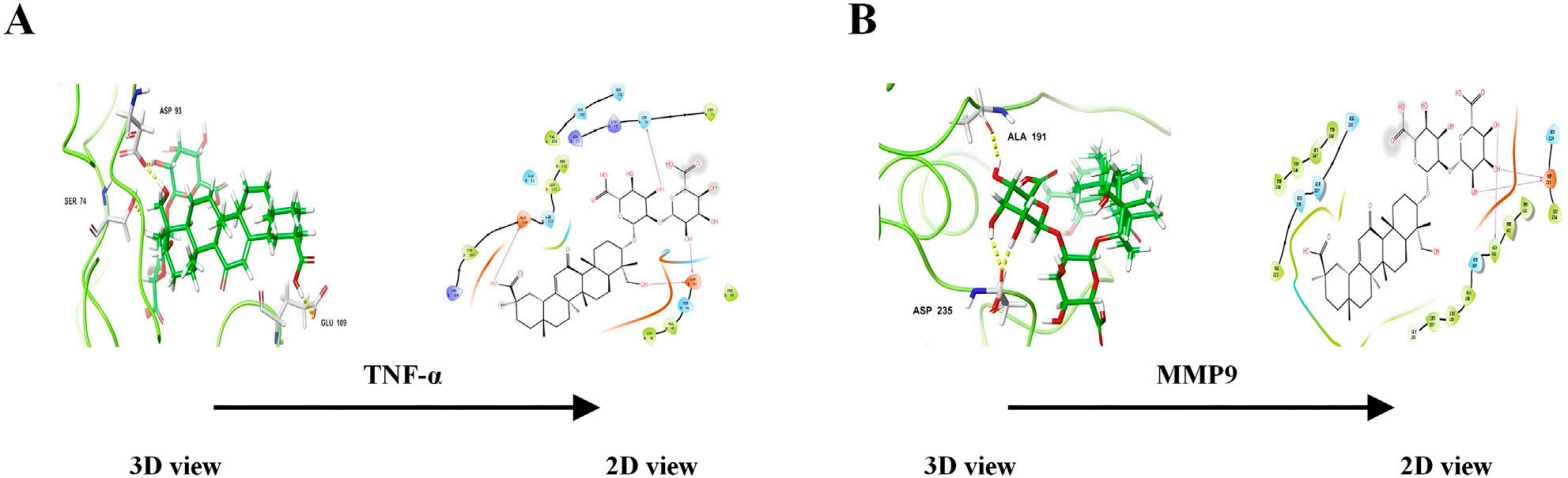
Molecular diagram of docking of metabolites.

Through network analysis and literature review, we identified two significant targets: TNF-α and MMP9. Specifically, LG2 forms hydrogen bonds with MMP9 at two amino acids, ALA-191 and ASP-235, both located near the active site. Similarly, LG2 establishes hydrogen bonds with TNF-α at three amino acids: GLU-109, SER-74, and ASP-93, all near the active site. These interactions are depicted in Figure 4. Docking studies of these targets with LG2 indicated that both TNF-α and MMP9 demonstrate strong binding affinity for LG2, as detailed in Table 2.

**TABLE 2:** Molecular docking results.

Active ingredient	Docking score (kcal/mol)	
	TNF-α	MMP9
Licoricesaponin G2	−4.849	−6.392

### LG2 protected against bleomycin-induced pulmonary fibrosis in mice

**FIGURE 5:**
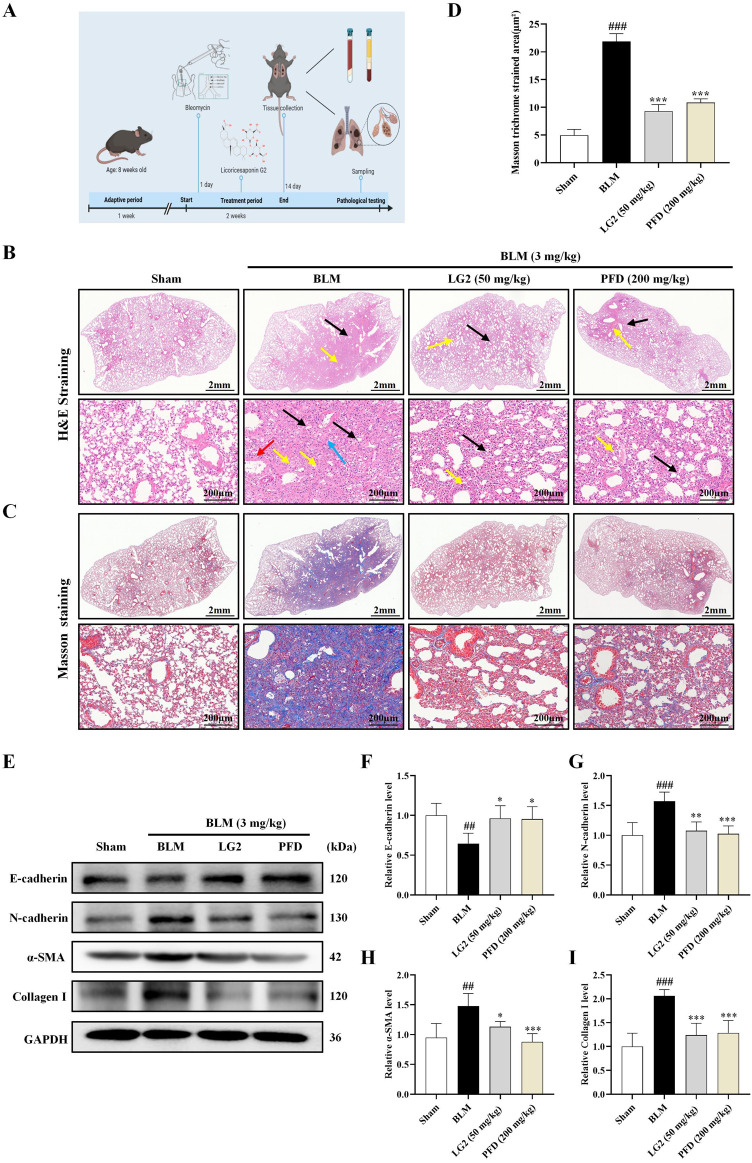
Evaluation of LG2’s effect on lung histology in C57 mice. **(A)** Workflow of the animal experiment. **(B)** Lung histology with H&E staining (arrows indicate: black—narrowed alveoli; yellow—lymphocyte/neutrophil infiltration; red—inflammatory cells surrounding blood vessels; blue—bronchial cell degeneration). **(C)** Masson’s trichrome staining of lung tissue. **(D)** Quantitative analysis of lung fibrosis (lower panel, scale bar = 200 μm, enlarged from upper panel, scale bar = 2 mm). **(E)** Western blot for protein expressions of E-cadherin, N-cadherin, α-SMA, and Collagen I in lung tissue. **(F–I)** Quantitative protein expression analysis using ImageJ software (n = 5). *p < 0.05, **p < 0.005, ***p < 0.001, compared to the BLM group. ^#^ *p* < 0.05, ^##^ *p* < 0.005, ^###^ *p* < 0.001, compared to the Sham group.

H&E staining revealed significant alterations in the lung tissues of mice induced by BLM alone, as shown in Figure 5B. Compared to the sham-operated group, lung tissues in the BLM group exhibited pronounced pathological changes including extensive connective tissue proliferation (indicated by black arrows), blurred alveolar structures, and widespread infiltration of lymphocytes and neutrophils (yellow arrows). Additionally, notable perivascular infiltration of inflammatory cells forming vascular sleeves was observed (red arrows), alongside cytoplasmic vacuolation in some bronchial epithelial cells (blue arrows). Following treatment with LG2 or pirfenidone (PFD), marked improvements in inflammation and fibrosis were observed in the lung tissues. Masson’s trichrome staining, shown in Figure 5C, further highlighted these changes; the blue-stained areas, indicative of collagen, pointed to the presence of fibrotic tissue. Compared to the sham group, there was a substantial increase in blue collagen fiber deposition in the lung tissues of the BLM group. However, this deposition was notably reduced after treatment, suggesting that LG2 effectively inhibits collagen fiber deposition in BLM-induced lung tissue in mice, as demonstrated in the quantitative analysis of Masson’s trichrome staining (Figure 5D). To further understand the molecular impact of LG2 on PF, we conducted western blot analyses. Compared to the sham group, the BLM group exhibited reduced E-cadherin protein expression and increased levels of N-cadherin, α-SMA, and Collagen I. Post-treatment, there was an upregulation of E-cadherin and a decrease in the other markers (Figures 5E–I). These findings suggest that LG2 treatment significantly alleviates the pathological damage and pulmonary fibrosis in the lung tissues of mice induced by BLM.

### LG2 inhibited EMT and ECM accumulation in mice lung tissues

**FIGURE 6:**
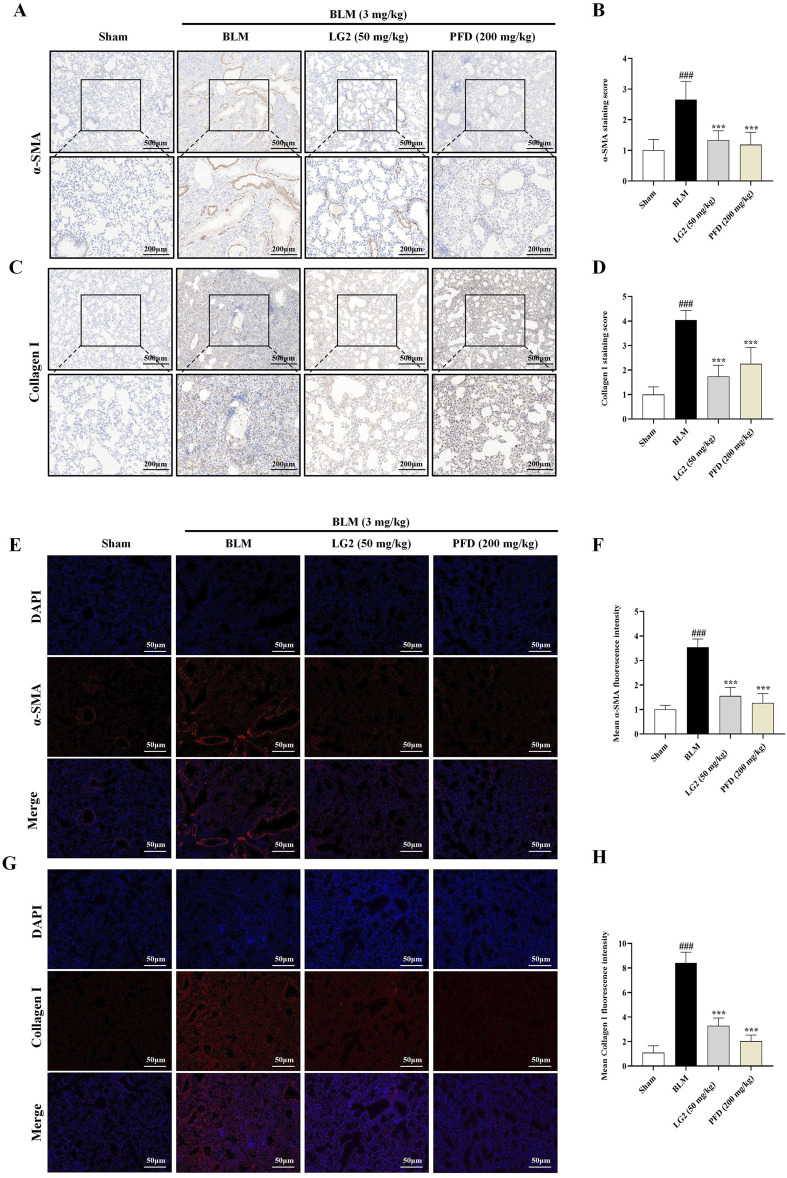
Assessment of LG2’s impact on EMT and ECM in mouse lung tissue using IHC and IF techniques. **(A)** IHC image of α-SMA. **(B)** Quantitative analysis of α-SMA protein expression. **(C)** IHC image of Collagen I. **(D)** Quantitative assessment of Collagen I protein expression (lower panel images, scale bar = 200 μm, enlarged from upper panel, scale bar = 500 μm). **(E)** IF image showing α-SMA distribution. **(F)** Quantitative analysis of α-SMA expression. **(G)** IF image showing Collagen I distribution. **(H)** Quantitative Collagen I expression analysis (scale bar = 50 μm) (n = 5). *p < 0.05, **p < 0.005, ***p < 0.001, compared to the BLM group. ^#^ *p* < 0.05, ^##^ *p* < 0.005, ^###^ *p* < 0.001, compared to the Sham group.

Our study investigated the roles of EMT and ECM in the development of PF, specifically examining the regulatory effects of LG2 on BLM -induced PF in mice. We employed IHC and IF to assess the levels of α-SMA and Collagen I proteins in lung tissues. The results demonstrated that in BLM-induced mice, there was an increase in the expression of α-SMA and Collagen I. However, following treatment with LG2 or PFD, the levels of both proteins significantly decreased, as shown in Figures 6A–H.

### Effect of LG2 on cell viability

**FIGURE 7:**
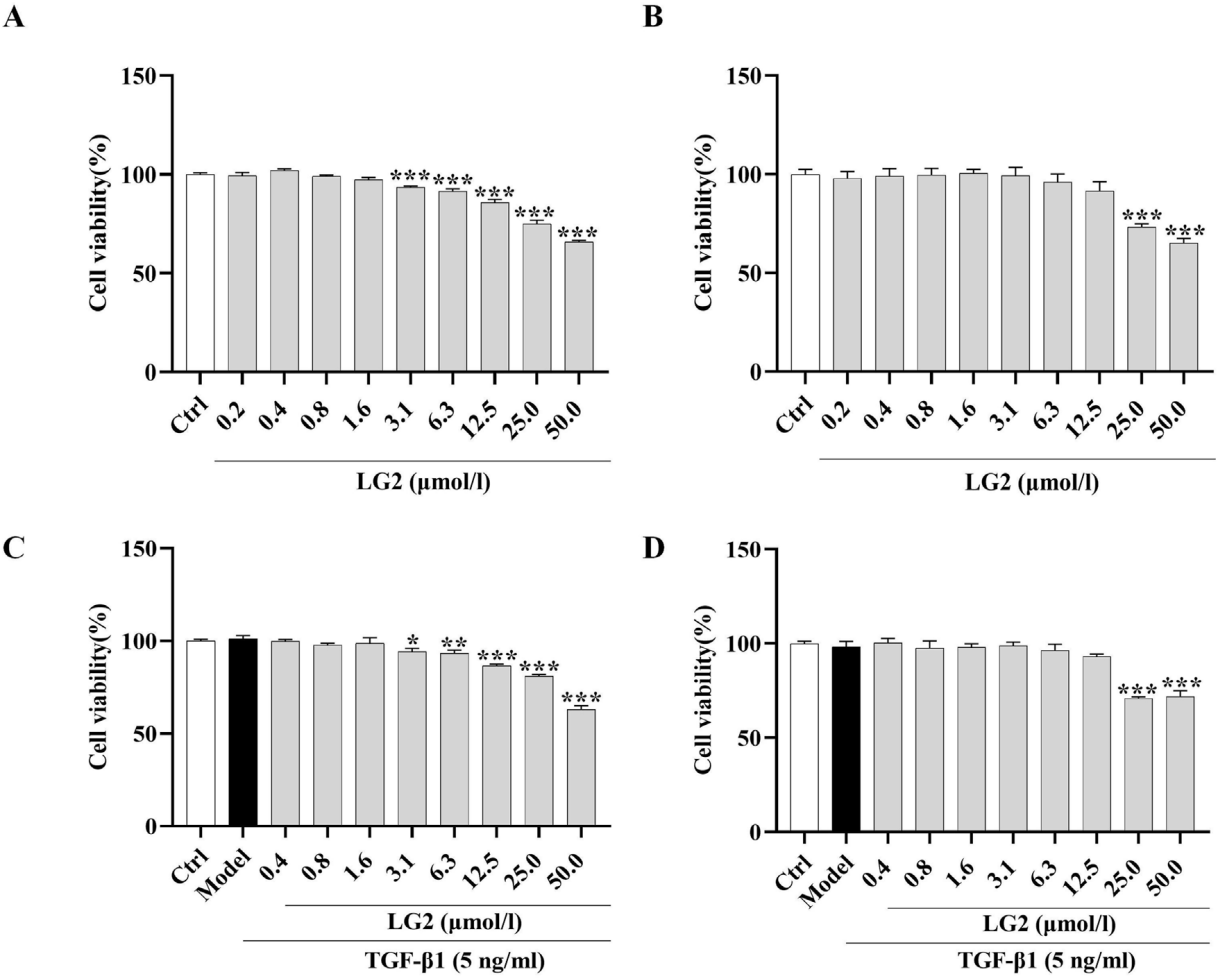
Effects of LG2 on cell viability in A549 and MRC5 cells. **(A,B)** Cytotoxicity of LG2 in A549 and MRC5 cells following 48-h treatment with various doses. **(C,D)** Impact of LG2 on cell viability in TGF-β1–induced A549 and MRC5 cells post 48-h treatment (n = 3).*p < 0.05, **p < 0.005, ***p < 0.001, compared to the Ctrl group (TGF-β1, 5 ng/mL).

TGF-β1 exerts a potent fibrotic effect and is known to induce EMT (Phan et al., 2021) and FMT (Zhang et al., 2021). These processes contribute to the formation of cells with mesenchymal characteristics, such as fibroblasts and myofibroblasts, which are central to the secretion of ECM and the progression of fibrosis (Sun et al., 2013). This rationale underpinned our choice of A549 and MRC5 cells for *in vitro* experiments to further explore the anti-PF mechanism of LG2 in inhibiting TGF-β1-induced transitions. Initially, we assessed the impact of varying concentrations of LG2 (0.2–50 μmol/L) on the viability of these cell types. Notably, compared to the control, A549 cells treated with concentrations of 3.1 μmol/L or higher for 48 h exhibited a significant reduction in viability (Figure 7A). Similarly, MRC5 cell viability showed a marked decrease at concentrations of 25 μmol/L under the same experimental conditions (Figure 7B). Additionally, the viability of A549 and MRC5 cells was assessed under treatment with TGF-β1 to simulate a fibrotic environment (Figures 7C, D). The therapeutic dosages used in subsequent experiments were determined based on these cytotoxicity assessments.

### LG2 alleviated TGF-β1-induced EMT/FMT and ECM accumulation in A549/MRC5 cells

**FIGURE 8:**
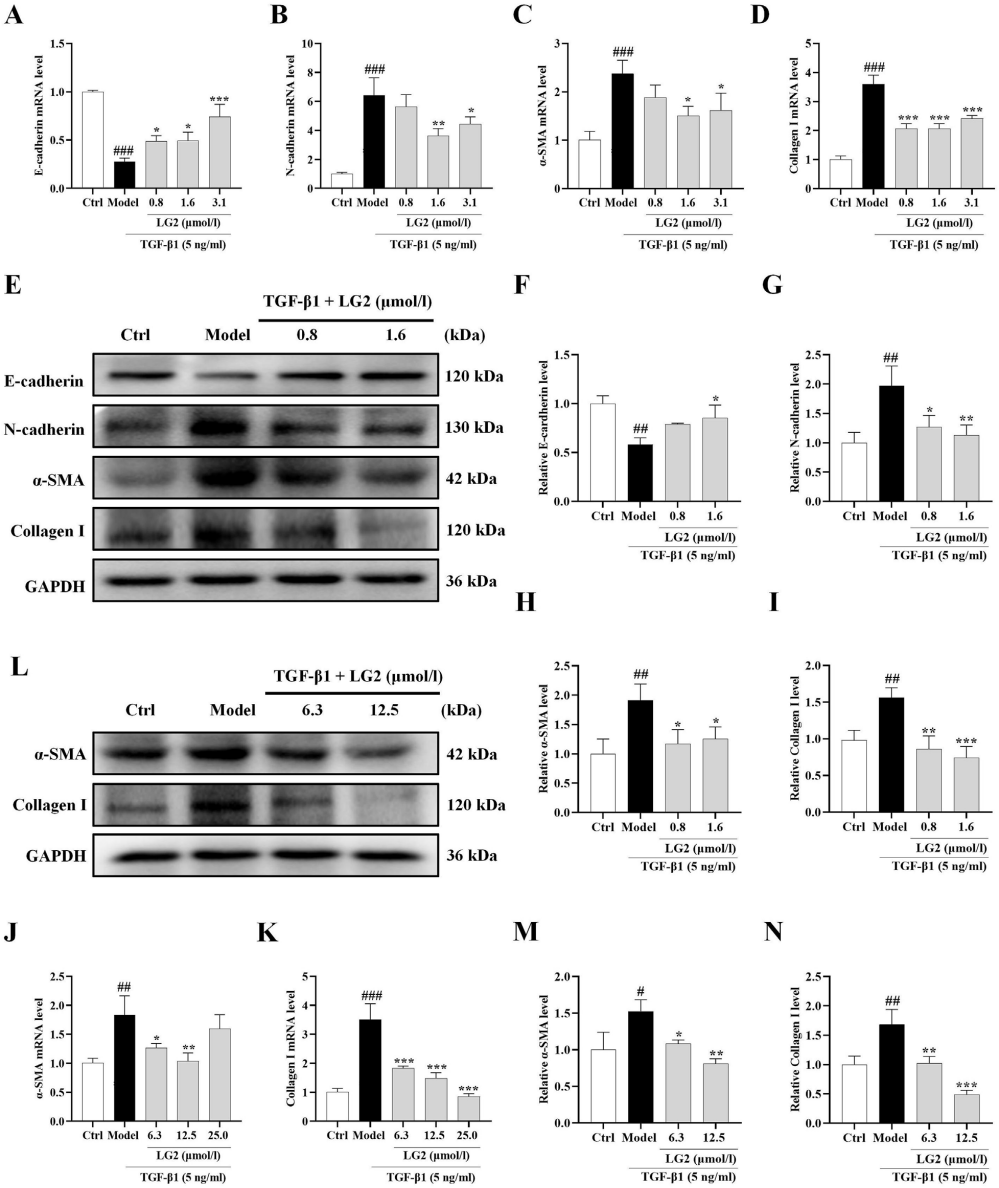
Impact of LG2 on EMT/FMT and ECM in A549 and MRC5 cells via qPCR and western blot analysis. (**A–D)** qPCR determination of mRNA levels of E-cadherin, N-cadherin, α-SMA, and Collagen I in A549 cells. **(E)** Western blot analysis of these proteins in A549 cells. **(F–I)** Quantification of protein expression using ImageJ software. **(J–K)** qPCR analysis of α-SMA and Collagen I mRNA levels in MRC5 cells. **(L)** Western blot analysis of these proteins in MRC5 cells. **(M,N)** Protein expression quantification using ImageJ software (n = 3). *p < 0.05, **p < 0.005, ***p < 0.001, compared to the Model group. ^#^ *p* < 0.05, ^##^ *p* < 0.005, ^###^ *p* < 0.001, compared to the Ctrl group.

We determined LG2 dosages using the MTT assay. Dosages of 0.8, 1.6, and 3.1 μmol/L were administered to A549 cells and 6.3, 12.5, and 25 μmol/L to MRC5 cells. After an incubation period of 48 h, qPCR analysis showed a decrease in mRNA levels of E-cadherin and an increase in N-cadherin, α-SMA, and collagen I in TGF-β1-stimulated A549 and MRC5 cells (Figures 8A–D, J, K). Subsequent to LG2 treatment, an increase in E-cadherin and a decrease in N-cadherin, α-SMA, and collagen I were observed. Western blot results confirmed these findings (Figures 8E–I, L–N). In summary, LG2 effectively mitigated the initiation of EMT and FMT in TGF-β1-stimulated A549 and MRC5 cells, facilitating ECM degradation. Consequently, we selected A549 cells for further investigation into LG2’s protective mechanisms against PF.

### LG2 inhibited TGF-β1-induced migration ability

**FIGURE 9:**
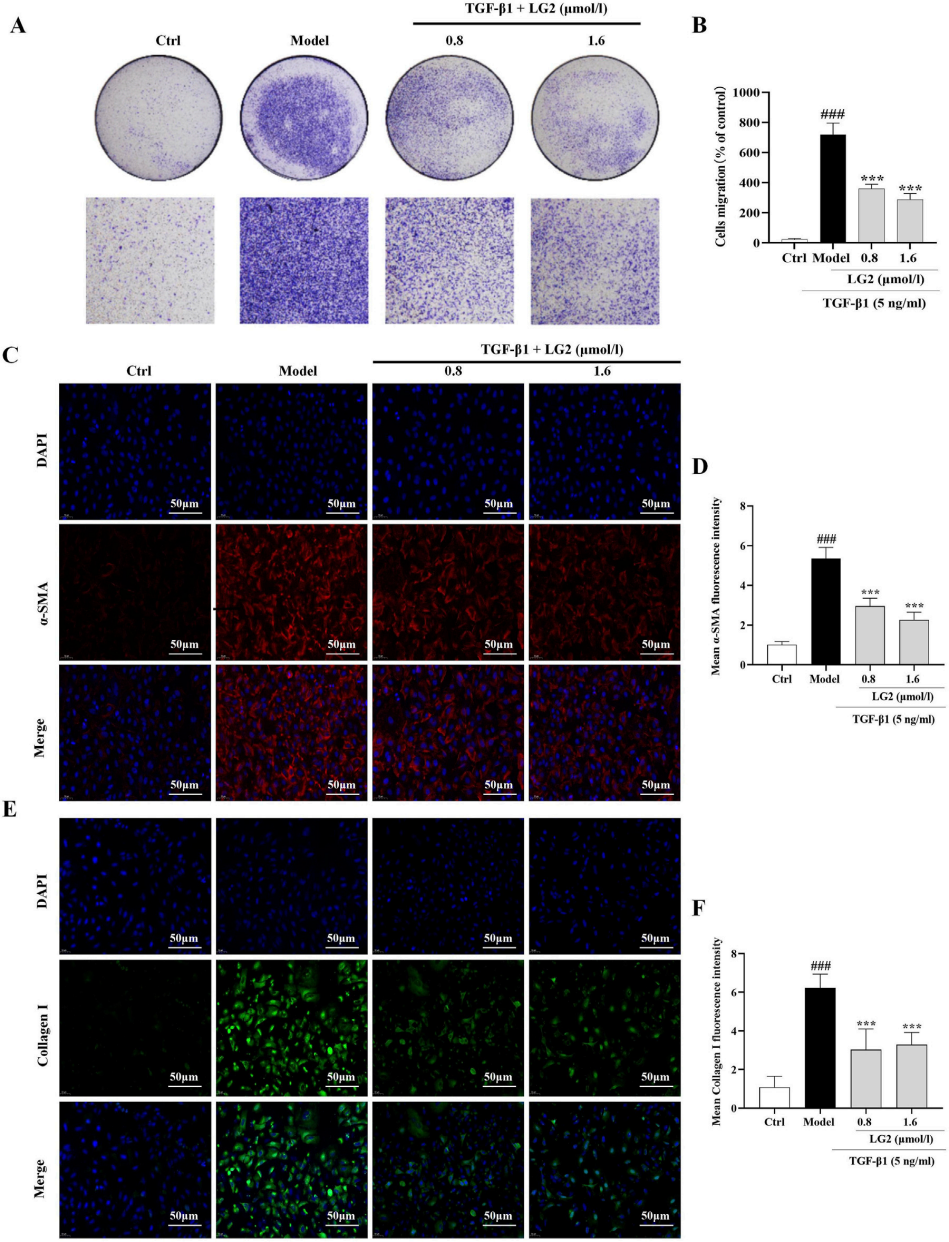
Inhibition of TGF-β1–induced migration in A549 cells by LG2. **(A)** Effects of LG2 on enhanced migration ability of A549 cells induced by TGF-β1, determined via a transwell migration assay. **(B)** Quantitative analysis using ImageJ software. **(C)** IF image showing α-SMA distribution. **(D)** Quantitative analysis of α-SMA expression. **(E)** IF image showing Collagen I distribution. **(F)** Quantitative analysis of Collagen I expression (scale bar = 50 μm) (n = 3). *p < 0.05, **p < 0.005, ***p < 0.001, compared to the Model group. ^#^ *p* < 0.05, ^##^ *p* < 0.005, ^###^ *p* < 0.001, compared to the Ctrl group.

A549 cells undergoing EMT exhibit decreased cell-cell adhesion and enhanced migratory capabilities (Xu et al., 2017). Our findings indicate that TGF-β1 significantly increases the migratory capacity of these cells. However, LG2 treatment notably inhibited this enhanced migration (Figures 9A, B). Additionally, we assessed the expression of α-SMA and collagen I using IF, as both proteins play crucial roles in cell transdifferentiation and migration. The results (Figures 9C–F) demonstrate that LG2 effectively reduced the elevated levels of α-SMA and collagen I induced by TGF-β1.

### Activation of TNF-α signaling pathway impaired the anti-fibrosis effects of LG2 *in vivo* and *in vitro*

**FIGURE 10:**
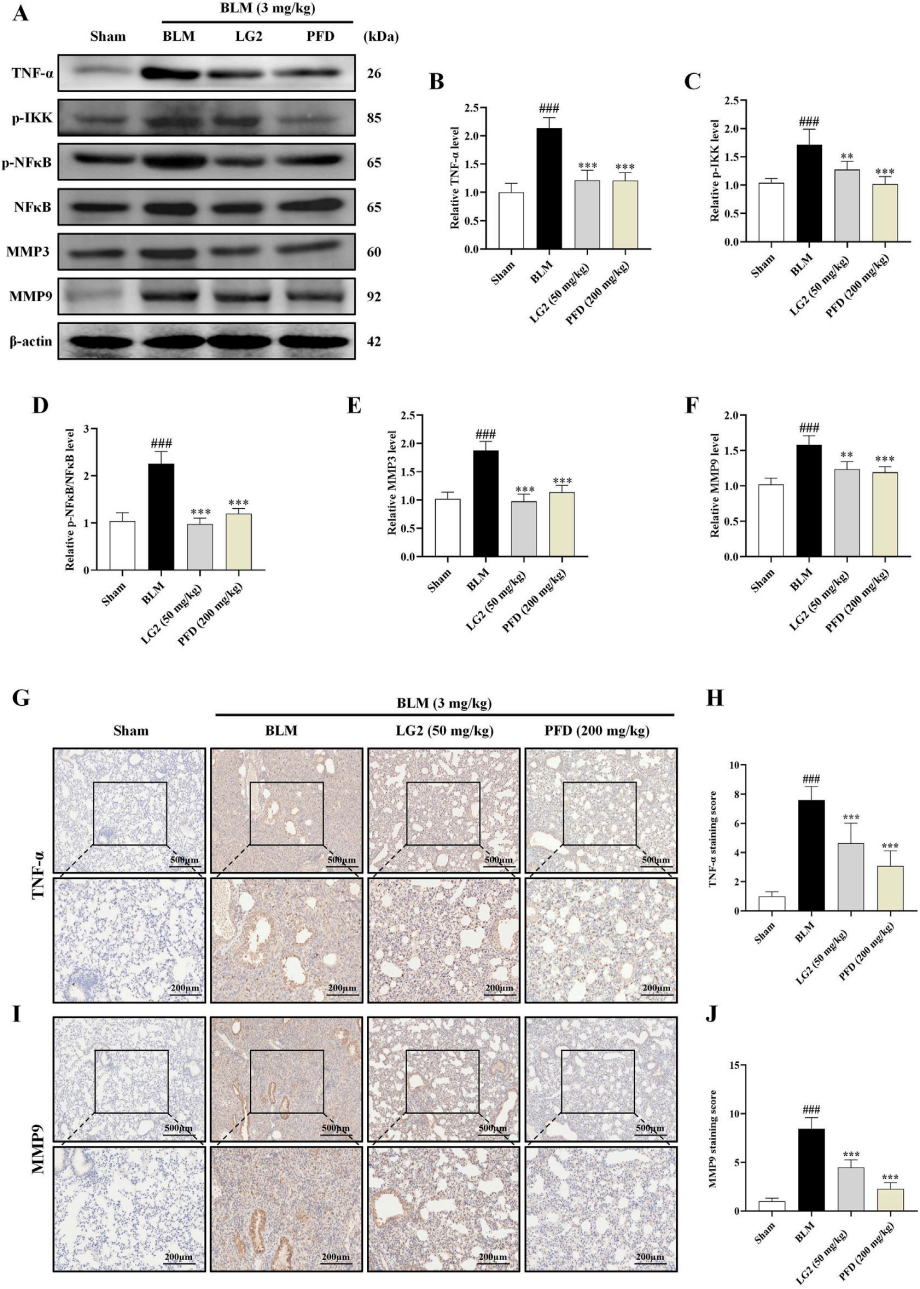
Inhibition of the TNF-α signaling pathway by LG2 in C57 mice: combined western blot and IHC analysis. **(A)** Western blot detection of TNF-α, p-IKK, p-NFB, NFκB, MMP3, and MMP9 proteins expression in lung tissue. **(B–F)** Quantitative analysis of proteins expression using ImageJ software. **(G)** IHC image of TNF-α. **(H)** Quantitative assessment of TNF-α protein expression. **(I)** IHC image of MMP9. **(J)** Quantitative assessment of MMP9 protein expression (lower panel images, scale bar = 200 μm, enlarged from upper panel, scale bar = 500 μm) (n = 5). *p < 0.05, **p < 0.005, ***p < 0.001, compared to the BLM group. ^#^ *p* < 0.05, ^##^ *p* < 0.005, ^###^ *p* < 0.001, compared to the Sham group.

**FIGURE 11:**
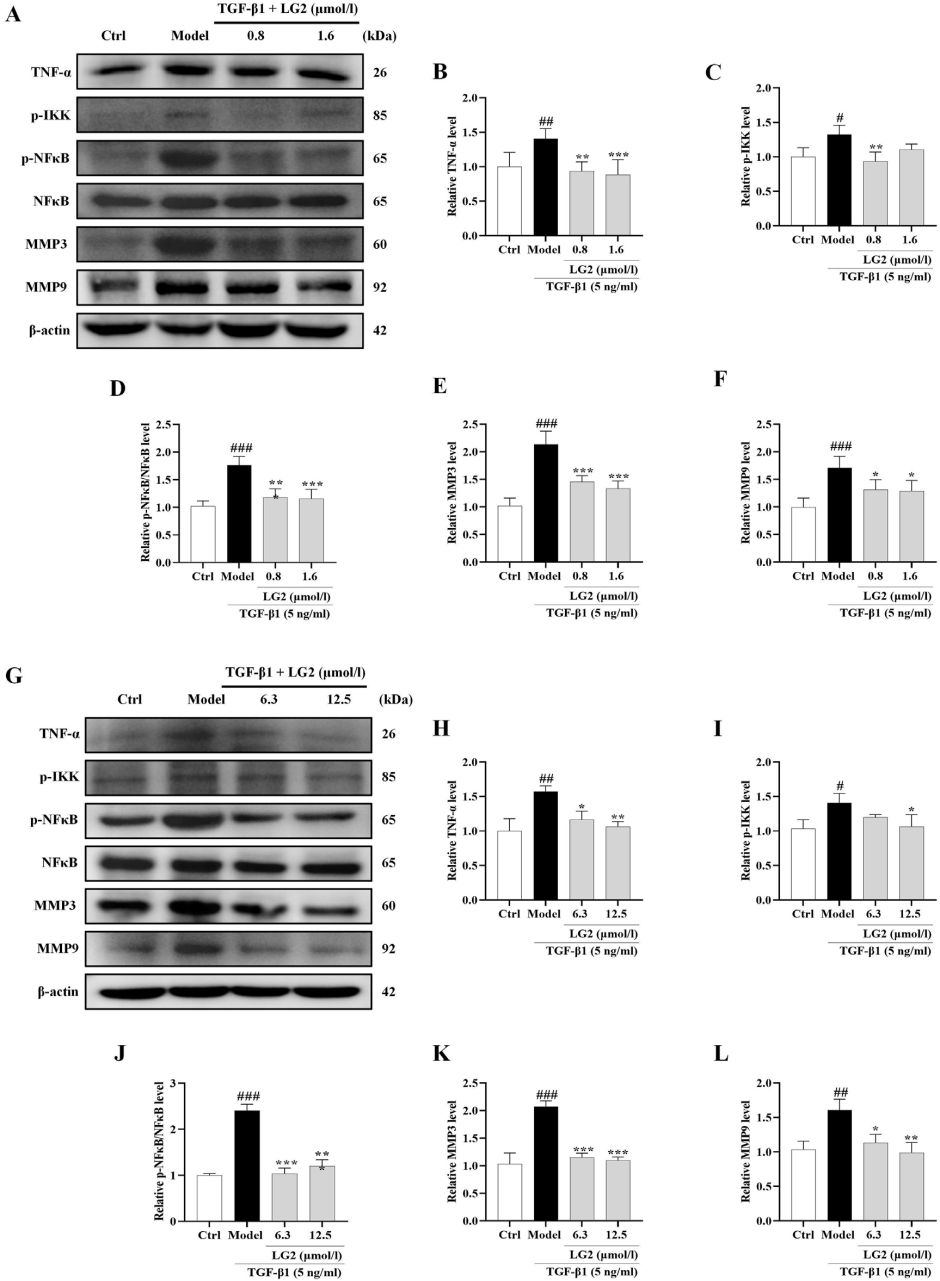
Inhibition of the TNF-α signaling pathway in A549 and MRC5 cells by LG2: western blot analysis. **(A)** Western blot detection of TNF-α, p-IKK, p-NFκB, NFκB, MMP3, and MMP9 proteins in A549 cells. **(B–F)** Quantitative analysis of proteins expression using ImageJ software. **(G)** Western blot detection of TNF-α, p-IKK, p-NFκB, NFκB, MMP3, and MMP9 proteins in MRC5 cells. **(H–L)** Quantitative analysis of proteins expression using ImageJ software. (n = 3). *p < 0.05, **p < 0.005, ***p < 0.001, compared to the Model group. ^#^ *p* < 0.05, ^##^ *p* < 0.005, ^###^ *p* < 0.001, compared to the Ctrl group.

**FIGURE 12:**
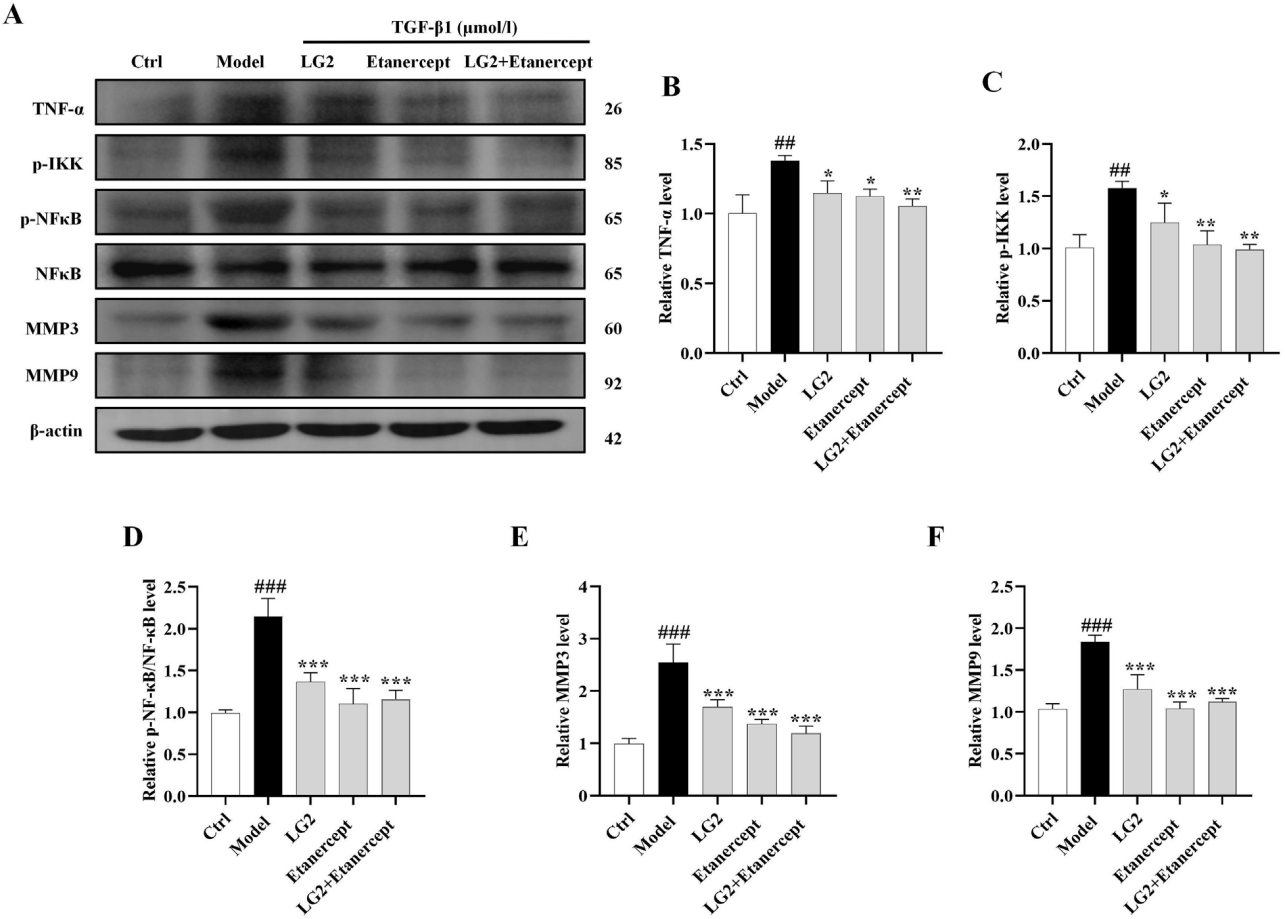
Verification of LG2 inhibiting the TNF-α signaling pathway: western blot analysis. **(A)** Western blot detection of TNF-α, p-IKK, p-NFκB, NFκB, MMP3, and MMP9 proteins in A549 cells after adding Etanercept. **(B–F)** Quantitative analysis of proteins expression using ImageJ software. (n = 3). *p < 0.05, **p < 0.005, ***p < 0.001, compared to the Model group. ^#^ *p* < 0.05, ^##^ *p* < 0.005, ^###^ *p* < 0.001, compared to the Ctrl group.

Having established the significant roles of EMT and ECM in the pathogenesis of PF, we further explored their internal mechanisms of action. Network analysis and molecular docking studies have indicated a close association between the development of PF and the TNF-α signaling pathway. To validate this hypothesis, we initially examined the expression levels of TNF-α, p-IKK, p-NFκB, NFκB, MMP3, and MMP9 using western blot analysis and IHC *in vivo* and *in vitro* (Figures 10, 11). As anticipated, compared to the control group, the model group exhibited increased protein levels of TNF-α, p-IKK, p-NFκB, MMP3, and MMP9. Following treatment, the expression levels of above proteins were significantly reduced following treatment with LG2. The TNF-α signaling cascade, known for its critical role in regulating inflammation. In order to further confirm the involvement of the TNF-α pathway in modulating EMT, we introduced the specific TNF-α inhibitor, etanercept, for experimental validation in TGF-β1-induced A549 cells. The cells were categorized into several groups: Ctrl, TGF-β1, TGF-β1+LG2 (1.6 μmol/L), TGF-β1+Etanercept (100 μg/mL) (Gong et al., 2021), and TGF-β1+LG2+Etanercept. Western blot results demonstrated that etanercept considerably reduced the expression of inflammatory proteins (Figures 12A–F). Notably, the simultaneous use of etanercept and LG2 further decreased the inflammatory levels. These results confirm that LG2 exerts anti-fibrosis effects by inhibiting the TNF-α signaling pathway, modulating EMT, and remodeling the ECM.

## Discussion

PF is a chronic, progressive, and potentially fatal connective tissue disease (Wijsenbeek and Cottin, 2020). The primary pathological mechanisms involved in the progression of PF include alveolar epithelial to mesenchymal transition (EMT) (Hewlett et al., 2018), migration, proliferation, and activation of fibroblasts, known as fibroblast to myofibroblast transition (FMT) (Wang et al., 2024), ECM deposition (Zhang et al., 2022), and formation of abnormal scar tissue (Sun et al., 2020). During this process, epithelial cells transform, losing their epithelial characteristics and apical-basal polarity, and acquiring mesenchymal properties. Recent studies have highlighted EMT as a primary initiator in the cascade leading to PF, suggesting that reducing EMT-related gene and protein marker expression could potentially reverse the progression of PF (Andugulapati et al., 2020). Additionally, the abnormal accumulation of myofibroblasts is a significant pathological feature in PF (Xie et al., 2016; Rock et al., 2011). Influenced by TGF-β1, fibroblasts differentiate into myofibroblasts, which then synthesize ECM components critical for lung tissue repair. However, chronic inflammatory stimuli can cause myofibroblasts to resist apoptosis, leading to abnormal wound healing and excessive ECM accumulation, which consequently reduces lung tissue elasticity and perpetuates a cycle of fibrosis (Upagupta et al., 2018). Gene silencing or targeting specific internal modifications to disrupt the myofibroblast transition has emerged as a promising therapeutic strategy (Zhang et al., 2021). Traditionally, alveolar epithelial cells and fibroblasts are considered key players in the development of PF (Wang et al., 2023), and accordingly, this study chose A549 and MRC5 cells as *in vitro* models.

In recent years, the therapeutic potential of natural TCM has attracted significant attention. Renowned researchers worldwide have acknowledged the substantial benefits of natural TCM and its metabolites in treating respiratory diseases. During the COVID-19 pandemic and its repercussions, natural TCM demonstrated unique advantages in medical practice. A multicenter, parallel-group, double-blind, randomized controlled trial showed that integrating natural TCM with conventional medicine can enhance treatment outcomes for PF caused by COVID-19. This combination not only improves patient prognosis and quality of life but also significantly extends the life expectancy of individuals with PF (Lu et al., 2021). Additionally, Chinese botanical drug have been identified as a rich source of diverse natural products, including flavonoids, polyphenols, terpenes, and alkaloids (Liu et al., 2021). Glycyrrhizic acid, an active metabolite derived from Glycyrrhiza uralensis Fisch (Gancao), can ameliorate liver fibrosis and inhibit hepatic stellate cell activation by enhancing the CUGBP1-mediated IFN-γ/STAT1/Smad7 pathways, indicating its potential as a preventive agent for liver fibrosis (Guo et al., 2023). The primary metabolites of Astragalus membranaceus (Fisch.) Bunge (Huangqi) and Glycyrrhiza uralensis Fisch (Gancao), namely, total astragalus saponins and glycyrrhizic acid, synergistically combat liver fibrosis via the TGF-β1/Smads pathway (Zhou et al., 2016). Thus, the multi-component, multi-target, and multi-pathway approach of natural TCM has become a distinctive feature in pharmaceutical research. Building on these insights, our study investigated the effects of the natural active metabolite LG2 in three experimental models: C57 mice, A549, and MRC5 cells. After 2 weeks of gavage treatment, a significant reduction in PF was observed. Histological analyses, including H&E and Masson staining, showed that LG2 treatment effectively reduced inflammation and collagen fiber deposition in mouse lung tissue. IHC and IF assessments of α-SMA and collagen I protein levels in lung tissues indicated that LG2 or pentachlorophenol (PCP) treatment could significantly decrease the expression of these proteins. At the molecular level, in BLM -induced C57 mice and TGF-β-induced A549 and MRC5 cells, our results demonstrated that LG2 decreased the overexpression of EMT/FMT and ECM markers (N-cadherin, α-SMA, and collagen I) and reinstated epithelial characteristics (E-cadherin). In conclusion, we have shown that LG2 can effectively intervene in and delay PF progression by mitigating the onset of EMT/FMT, reducing ECM deposition, and enhancing ECM degradation.

This study adopted network analysis to explore the potential mechanisms through which LG2 might prevent PF. Utilizing databases such as Drugbank, SwissTargetPrediction, ZINC, and PharmMapper, we identified 175 potential targets of LG2. Through GeneCards, OMIM, and PharmGKB, we pinpointed 6,119 PF-related targets. Venn diagram analysis then revealed 127 intersecting targets of LG2 for PF. The PPI network, constructed using the STRING database, highlighted key targets including VEGFA, SRC, PTGS2, JUN, MMP9, CASP3, STAT3, IL2, FGF2, and PTPRC. These targets are implicated in various pathological processes such as the inflammatory response (Hou et al., 2018), ECM deposition, EMT, and fibroblast differentiation (Espindola et al., 2021), all critical in the onset of PF. GO biological process and KEGG pathway enrichment analyses further suggested that LG2 might affect cell migration and proliferation, potentially modulating numerous PF-related signaling pathways. Molecular binding energy analysis also indicated that LG2 might inhibit the TNF-α and MMP9 proteins, thereby influencing the TNF-α pathway. However, beyond the TNF-α signaling pathway, the roles of other pathways targeted by LG2 require further exploration.

**FIGURE 13:**
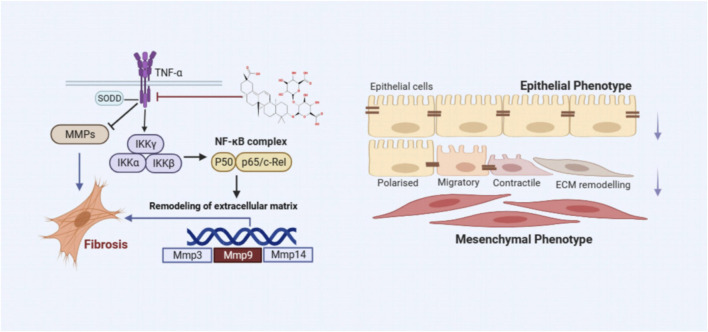
The scheme demonstrating the molecular mechanism underlying the effects of LG2 in preventing and treating PF.

Inflammation is widely recognized as a cornerstone in the pathogenesis of PF. During the early stages of PF, recruited inflammatory cells release numerous mediators that foster interactions between epithelial cells, EMT, and adjacent mesenchymal cells, as depicted in Figure 13 (Hewlett et al., 2018). A defining feature of PF is the excessive deposition of the ECM, indicating irreversible changes in lung tissue. Chronic inflammatory stimuli can alter the expression of MMPs, enzymes essential for ECM degradation (De Almeida et al., 2022). An imbalance between inflammatory factors and MMPs can lead to ECM dysregulation, facilitating smooth muscle cell proliferation, fibroblast activation, and collagen accumulation. Elevated α-SMA expression signifies fibroblast activation and their transformation into myofibroblasts, which contributes to excessive ECM deposition (Wynn, 2011). Over time, this can result in the destruction of lung tissue structure, impairing gas exchange and ultimately leading to PF. Under physiological conditions, MMPs not only exhibit proteolytic functions but are also involved in processing and activating ECM-related proteins, regulating leukocyte function, antibacterial defense, and cell migration (Parks et al., 2004). Therefore, MMPs can either promote or inhibit PF progression, depending on the context. Alongside the TNF-α signaling pathway identified through network analysis, this work also assessed TNF-α, p-IKK, p-NFκB, NFκB, MMP3, and MMP9 protein expressions *in vivo* and *in vitro* using western blot and IHC techniques. The results indicated that LG2 treatment reduced the expression of above proteins, suggesting that LG2 could mitigate PF by inhibiting the TNF-α signaling pathway. Additionally, molecular docking revealed promising interactions between TNF-α, MMP9, and the natural metabolite LG2. This finding aligns with our research objectives, highlighting the anti-inflammatory and antioxidative pharmacological benefits of LG2 via the TNF-α pathway, potentially suppressing inflammatory cytokine secretion and reducing cellular damage during early PF development.

It has been proven that the natural bioactive metabolites from GC, in particular, Licoricesaponin G2 (LG2), the biological effects of which appear to involve the regulation of modulating EMT, remodeling the ECM, and inflammation so far, has shown the potential therapeutic benefits for PF. This study not only has important theoretical implications but also unveils a novel natural metabolite and molecular mechanism for treating PF. LG2 is a monomer composition from GC, we need to provide enough evidence to prove its anti-fibrosis effects in the animal and/or cell models in the future. Therefore, more relevant studies should be undertaken to clarify the others targets and mechanisms, the research progress and viewpoint provided in this study will be valuable for future research of LG2 in the development of PF drug. Meanwhile, we hope to provide more research achievements on LG2 in subsequent experimental research.

## Conclusion

By leveraging network analysis, molecular docking, and experimental verification methods, we identified that LG2 can modulate the initiation of EMT and FMT, as well as ECM degradation, by inhibiting the TNF-α signaling pathway. This intervention potentially delays the progression of PF. Our research results indicate that LG2, as a novel natural active product not currently reported in the treatment of PF, warrants further exploration.

## Figures and Tables

**Table:** 

**GC**	Glycyrrhiza uralensis Fisch
**TCM**	Traditional Chinese Medicine
**PF**	Pulmonary fibrosis
**LG2**	Licoricesaponin G2
**MTT**	3-(4,5-dimethylthiazol-2-yl)-2,5-diphenyltetrazolium bromide
**qPCR**	Quantitative Real-Time PCR
**A549**	Human lung carcinoma cells
**MRC5**	Human fetal lung fibroblast cells
**BLM**	Bleomycin
**FBS**	Fetal bovine serum
**TNF-α**	Tumor necrosis factor-alpha
**MMP9**	Matrix metallopeptidase 9
**EMT**	Epithelial-mesenchymal transition
**ECM**	Extracellular matrix
**FMT**	Fibroblast-myofibroblast transition
**Danshen**	Salvia miltiorrhiza Bunge
**Huangqi**	Astragalus membranaceus (Fisch.) Bunge
**COVID-19**	Coronavirus disease 2019
**UHPLC/IM-QTOF-MS**	Ultra-high performance liquid chromatography/ion mobility-quadrupole time-of-flight mass spectrometry
**TGF-β1**	Transforming growth factor β1
**TGF-βR1**	Transforming growth factor β receptor type 1
**α-SMA**	alpha-smooth muscle actin
**Collagen I**	Collagen type I
**RIPA**	Radioimmunoprecipitation assay
**DMSO**	Dimethyl sulfoxide
**OMIM**	Online Mendelian Inheritance in Man
**PPI**	Protein-protein interaction
**BC**	Betweenness centrality
**CC**	Closeness centrality
**DC**	Degree centrality
**CM**	Cellular metabolite
**MF**	Molecular function
**BP**	Biological process
**GO**	Gene Ontology
**KEGG**	Kyoto Encyclopedia of Genes and Genomes
**PDB**	Protein data bank
**IF**	Immunofluorescent
**IHC**	Immunohistochemistry
**SDS-PAGE**	Sodium dodecyl sulfate polyacrylamide gel electrophoresis
**PVDF**	Polyvinylidene fluoride
**PBS**	Phosphate buffered saline
**BSA**	Bovine serum albumin
**VEGFA**	Vascular endothelial growth factor-A
**SRC**	Steroid receptor coactivator
**PTGS2**	Prostaglandin-endoperoxide synthase 2
**CASP3**	Caspase-3
**STAT3**	Signal transducer and activator of transcription 3
**IL-2**	Interleukin 2
**FGF2**	Fibroblast growth factor 2
**PTPRC**	Protein tyrosine phosphatase receptor type C
**ERK1**	Extracellular signal-regulated kinase 1
**ERK2**	Extracellular signal-regulated kinase 2
**MAPK**	Mitogen-activated protein kinase
**EGFR**	Epidermal growth factor receptor
**MMPs**	Matrix metalloproteinases.

## Data Availability

The original contributions presented in the study are included in the article/supplementary material, further inquiries can be directed to the corresponding authors.
